# Discover Internet of Things editorial, inaugural issue: Welcome from Editor-in-Chief

**DOI:** 10.1007/s43926-021-00007-6

**Published:** 2021-02-24

**Authors:** Ishfaq Ahmad

**Affiliations:** Computer Science & Engineering, University of Texas at Arlington, Arlington, TX USA

The current times are perhaps among the toughest periods in modern history. The Covid-19 pandemic has deeply affected the world’s population, affecting its way of life and impacting the economy as well as social priorities. There have been a staggering number of deaths and the burden on healthcare services is unprecedented, forcing us to adjust our ways of living, work and socialization. However, the Covid-19 pandemic, despite all the devastation it has wreaked, carries several important lessons. It may be a rude awakening, calling for greater connectedness, increased responsibility and more peace and harmony. Another remarkable cue left by this pandemic is that the role of computing has become more crucial than ever. We must be technologically more prepared to preemptively thwart similar or worse hazards in the future. While computing in general continues to make technological strides at an unprecedented pace, the COVID-19 virus is forcing us to switch our focus, redefine our priorities and realign our scientific and engineering directions. More than ever before, we have come to recognize the importance of computer-assisted collection, analysis, and dissemination of sensory data. We also need to invent means of remote healthcare and delivery of medicine and vaccines. Healthcare will be the most important consumer of IoT enabled technologies. Thus, IOT enabled infrastructure must be developed for healthcare and it will be crucial.

The importance of IoT-enabled technologies is no less profound in other sectors. As well as the vast amount of fresh knowledge that is emerging from the analysis of big data, new ubiquitous applications for previously unconceived scenarios are also evolving at an explosive rate. In fact, the IoT revolution is transforming the entire spectrum of computing-based services. A new wave of rapid infrastructure development is sweeping the globe, encompassing virtually everything digital ranging from data centers, supercomputers, clusters, embedded systems, servers, and networks to power grids, sensors, devices, appliances, mobile devices, and instruments. The IoT paradigm makes physical objects with sensing capabilities, including those with tags, smart meters, consumer electronic devices, health sensors and devices, mobile objects such as smart phones, drones, and vehicles, and home appliances such as microwaves, dishwashers, and televisions, a part of the Internet environment.

New IoT infrastructures, enabled by the already ever-present Internet technology, are finding new means of making the world a connected place. According to statistica.com, over 10 billion devices will exist by 2022. Projections by Ericson estimate that by 2025, there will be an installed base of 75.44 billion IoT connected devices worldwide, a fivefold increase in 10 years. Worldwide spending on the IoT reached $745 billion in 2019, an increase of 15.4% over the $646 billion spent in 2018, according to research done by the International Data Corporation (IDC). Moreover, the IDC expects worldwide IoT spending to surpass the $1 trillion mark in 2022. Thus, IoT-based technologies may serve as a critical engine driving the new economy.

IoT systems are being deployed in many capacities. Apart from healthcare, there is a rapid proliferation of smart homes, smart cities, offices, factories, etc. In bringing people and technology even closer, the IoT revolution is poised to lead us to an era wherein cyber, physical, social, and psychological spaces are integrated with greater harmony. This implies more natural machine-human interfaces, greater access to information, and improved decision making.

This staggering technological expansion brings with it profound economic and social implications as well as promising research opportunities and challenges. New research directions, development of knowledgebase, and training a new IT workforce are imminently desirable. IoT applications generate massive amounts of data in a variety of formats. Storage and analysis of the data require efficient, effective and secure data analytics algorithms. Tools using machine learning and other advanced techniques are required. In cloud-oriented IoT systems, sensory data is extracted, accumulated, and processed at cloud data centers, leading to prohibitively high latencies. Fog/edge computing circumvents this problem by providing real-time support through efficient utilization of proximity based computational resources across the IoT layers such as gateways, cloudlets, and switches/routers. To support evolving IoT applications, large-centralized cloud computing infrastructures are migrating to micro datacenters located at the network’s edge. This is leading to the escalation of the so called fog and edge computing paradigms that aim to utilize edge resources to off load computation which would normally have been carried out at the cloud data center to a resource that is closer to users or edge devices. Fog/Edge computing aims to improve the agility of cloud service deployments in addition to bringing computing resources closer to end-users.

In the realm of transportation, communication via IoT-connected automated vehicles is poised to be a game changer for the transportation industry. Almost all sorts of vehicles, ranging from automated trucks to buses and shared passenger cars, are expected to benefit from such applications at various scales. In most applications, vehicles can decide their maneuvers and services using their on-board sensors, such as cameras, and radars. However, their surrounding environments cannot be discovered by their on-board sensors alone. Their efficient passage requires not only current traffic support substructures (such as, road-side message boards, traffic lights etc.), but also advanced communication mechanisms providing access to vast and dynamic information. These can help to mitigate traffic bottlenecks, reduce fuel costs, and enhance environmental sustainability.

In sports and entertainment, we are witnessing innovating gaming mechanisms, delivery of video and music as well as new means entertainment. Similarly, in commerce and trade, we want the results of complex data analytics (e.g. real-estate, stock market and other investment tools) available in real-time on our cell phones. IoT-based applications are sorely needed in agriculture, manufacturing, wearable devices, energy and power, and driverless vehicles. The issue of data privacy and security, of course, must be an integral component these infrastructures.

The emergence of IoT coupled with fog/edge computing is leading to considerably greater computer system development. This includes engineering of scalable architectures, moving from closed systems to open systems, new operating systems, middleware, communication protocols, autonomic management, and addressing privacy and ethical issues involved in data sensing, storage, processing, and decision-making. Machine learning, deep learning, and artificial intelligence techniques are being widely explored for enhancing the scalability, reliability, and quality-of-service in the IoT applications. New extensions are required for current programming models that will allow developers to conceive and design novel applications to take advantage of the new paradigms. These challenges are prompting researchers to create knowledge that requires new avenues of publishing, sharing, and dissemination.

*Discover IoT* is among the Discover Journals series by Springer, which is a collection of fully open access journals committed to providing all authors a streamlined submission process, rapid review and publication, and a high level of author service at every stage. This new Series of OA journals from Springer Nature was announced in June 2020. The series will consist of up to 40 new titles covering hot topics from across the full range of applied science, physical, life, medical and social disciplines. More information about the Discover Series can be found here: www.springer.com/gp/campaign/discover-journals.

*Discover IoT* aims to publish original research papers in a variety of areas pertinent to IoT. The goal is to take advantage of new ideas about online data sharing and efficient publication tools designed to promptly present to its readers the state-of-the-art, addressing all parts of the value chain from concepts at the component, software, and system level as well as programming, operating systems, applications and other technology-oriented research topics. *Discover IoT* will publish a wide range of papers, survey articles, and comments that deal with all research areas of importance to experts, researchers, teachers, practitioners, engineers, and students. Thus, the journal will enable research communities across the globe to share their findings and ideas in the fast expanding field of IoT.

I am pleased to state that *Discover IoT* has gathered a very prestigious editorial board, with members hailing from around the world. In particular, one can notice that *Discover IoT* is very supportive of diversity (race, country, gender, etc.). There are six Associate Editors, representing six sub-sections (indicated in the parenthesis) given as follows:

- Barbara Carminati, University of Insubria, Italy (IoT Systems);
- Manfred Huber, The University of Texas at Arlington, USA (IoT-Enabled AI);
- Pradeep Kumar Singh, ABES Engineering College, India (IoT Applications);
- Fatemeh Tehranipoor, The Santa Clara University, USA (IoT Devices);
- Bhavani Thuraisingham, The University of Texas at Dallas, USA (Security and Privacy);
- Zheng Xu, Shanghai University of Medicine & Health Sciences, China (IoT Analytics).

Our editorial board also includes editors who are well-known researchers. I am thankful to all section editors and editorial board members for their willingness and commitment to serve the editorial mission of *Discover IoT*. The list of colleagues who have joined us in this endeavor is available at https://www.springer.com/journal/43926/editors.

In addition, we are honored to have an advisory board, which comprises of highly respected researchers and academicians from around the world. They are:

- Vipin Kumar, University of Minnesota, USA;
- Geoffrey Fox, Indiana University, USA;
- Gurdip Singh, Syracuse University, National Science Foundation, USA;
- C.-C. Jay Kuo, University of Southern California, USA;
- Chang Wan Chen, Chinese University of Hong Kong, Shenzhen, China;
- Kang Wang, University of California at Los Angeles, USA;
- Ashraf Kassim, Singapore University of Technology and Design, Singapore;
- Jack Dongarra, University of Tennessee at Knoxville, USA.

I am highly thankful to the *Discover IoT* staff, especially Sweater Shi, and the Springer Management. I sincerely thank Toby Charkin, Publishing Director at Springer, for recognizing the potential of this journal and for providing the necessary support to turn it into a reality.

Maintaining a high standard of paper review and quality and being at the forefront of scientific discovery and innovation are the prime aims of *Discover IoT*. As such, we will need to reach several milestones, including publishing high-quality papers with a rapid turnaround time, supporting cutting-edge research topics through topical issues, and encouraging multi-disciplinary themes.

*Discover IoT* is off to a good start. We have regular papers under review, and six topical collections are currently planned (while more are in the pipeline). The six collections are:

- Cyber-Physical Systems Services and Technologies for Smart-Health;
- Big Data Applications and Techniques in Cyber Intelligence for Internet of Things;
- Emerging IoT Applications for Future Smart Cities;
- Emerging Physical Layer Security Solutions for IoT Devices;
- Blockchain with IoT;
- Enabling AI Technologies for Megadata Mining in the Internet of Cognitive Things (SI-AIoCT).

The papers in the inaugural issue, published as a topical collection, were submitted by invitation. They were authored by leading experts in the field. We have five invited papers from these world-class researchers. A brief description of these follows:

- “Intelligent IoT Systems for Civil Infrastructure Monitoring: A Research Roadmap,” authored by Elisa Bertino, Mohammad Jahanshahi, Ankush Singla, and Rih-Teng Wu, provides a details perspective on using AI for IoT-based civil infrastructures [1].
- “QoE in IoT: A Vision, Survey and Future Directions,” authored by Kaneez Fizza, Abhik Banerjee, Karan Mitra, Prem Prakash Jayaraman, Rajiv Ranjan, Pankesh Patel, and Dimitrios Georgakopoulos, addresses the notion of Quality-of-Experience, which can be a key factor for quality control and decision making in autonomic IoT applications [2].
- “Swarm-based Counter UAV Defense System,” authored by Matthias Brust, Grégoire Danoy, Daniel Stolfi, and Pascal Bouvry. It gives a comprehensive overview of how to design a safety- and security-ensuring network of unmanned aerial vehicles [3].
- ““Chatty Devices” and Edge-based Activity Classification,” authored by Mike Lakoju, Amir Javed, Omer Rana, Peter Burnap, Samuelson Atiba, and Soumaya Cherkaoui, discusses human-man robot interaction in a manufacturing environment, touching upon critical research challenges [4].
- “Things in the Air: Tagging Wearable IoT Information on Drone Videos,” authored by Lan-Da Van, Ling-Yan Zhang, Chun-Hao Chang, Kit-Lun Tong, Kun-Ru Wu, and Yu-Chee Tseng, describes a surveillance system which employs drones that interact with wearable IoT devices for person identification. We expect to add one or two more papers in the inaugural issue [5].

I hope that *Discover IoT* develops into a community where we realize and acknowledge the progress of knowledge in the IoT revolution. Let us work together on growing and strengthening this community for sharing new knowledge. Please feel free to contact us if you have any thoughts or suggestions that can help us do our job better.
